# PseuSeal technique: endovascular repair of Iatrogenic pseudoaneurysm using ExoSeal

**DOI:** 10.1186/s42155-025-00536-z

**Published:** 2025-03-17

**Authors:** Takuya Haraguchi, Yuhei Kasai, Masanaga Tsujimoto, Yoshifumi Kashima, Katsuhiko Sato, Tsutomu Fujita

**Affiliations:** Department of Cardiology, Asia Medical Group, Sapporo Heart Center, Sapporo Cardio Vascular Clinic, Sapporo, Japan

**Keywords:** Pseudoaneurysm, Vascular closure device, Endovascular therapy, Percutaneous coronary intervention, Peripheral artery disease

## Abstract

**Introduction:**

The ExoSeal^®^ (Cordis, Florida, USA) is a bioabsorbable vascular closure device that facilitates hemostasis by deploying a polyglycolic acid (PGA) plug. This report presents the "PseuSeal technique," a novel approach to seal pseudoaneurysm using ExoSeal in an off-label manner.

**Methods:**

The PseuSeal technique includes the PseuSeal Snare and PseuSeal Rendezvous, both performed via a contralateral crossover approach. The choice of approach depends on whether a 4-Fr catheter can be advanced into the pseudoaneurysm cavity. If feasible, the PseuSeal Snare is selected; otherwise, the PseuSeal Rendezvous is employed. In the PseuSeal Snare, a snare is deployed from a 4-Fr catheter within the pseudoaneurysm cavity. An 18-gauge needle is then used to retrogradely puncture the snare loop. A 0.035-inch guidewire is passed through the needle lumen, captured by the snare, and externalized. In the PseuSeal Rendezvous, an 18-gauge needle retrogradely punctures the guidewire within the cavity, and the guidewire is advanced into the needle lumen for externalization. After externalizing the guidewire in both approaches, an ExoSeal-specific sheath is inserted over the guidewire through the pseudoaneurysm neck into the main vessel. A balloon is advanced from the crossover sheath into the main trunk to cover the pseudoaneurysm ostium. The ExoSeal system is then inserted through the second sheath. As the ExoSeal's indicator wire is withdrawn, the balloon is inflated to stabilize the indicator wire, ensuring precise deployment of the PGA plug at the pseudoaneurysm neck. Balloon inflation is maintained for 5 minutes, with an additional 5 minutes if necessary. Hemostasis is confirmed by angiography.

**Results:**

The PseuSeal technique was successfully applied in five common femoral artery　pseudoaneurysms, all of which had failed ultrasound-guided compression. Each case was treated using a 6-Fr ExoSeal device, with no complications or recurrences observed during follow-up. Case 1 involved an 87-year-old female who developed a pseudoaneurysm following a peripheral intervention. The PseuSeal Snare achieved hemostasis in 32 minutes. Case 2 involved a 60-year-old male presenting with a pseudoaneurysm after a coronary intervention. The PseuSeal Rendezvous achieved hemostasis in 50 minutes.

**Conclusion:**

The PseuSeal technique provides an effective alternative for pseudoaneurysm closure.

**Supplementary Information:**

The online version contains supplementary material available at 10.1186/s42155-025-00536-z.

## Introduction

The incidence of pseudoaneurysm following interventional procedures is as high as 7.7% [1]. The use of antiplatelet and anticoagulant therapies significantly increases the risk of pseudoaneurysm formation and complicates hemostasis. Ultrasound-guided compression (UGC) and thrombin injection (UGTI) have demonstrated success rates of 75% and 96%, respectively [2]. However, UGTI carries a risk of complications, such as distal embolization, and the recurrence rate of pseudoaneurysm after UGTI ranges from 6% to 14% [2].

The ExoSeal^®^ (Cordis, Florida, USA) is a bioabsorbable vascular closure device that promotes hemostasis by deploying a polyglycolic acid (PGA) plug externally over the arterial puncture site. The plug is absorbed within approximately three months. The deployment process involves inserting the ExoSeal device through the sheath, retracting the indicator wire at the device's tip to align it with the puncture site, and deploying the PGA plug onto the artery.

This report presents a novel therapeutic approach, the "PseuSeal technique," in which the ExoSeal plug is directly deployed at the aneurysmal neck to seal the pseudoaneurysm in an off-label use.

## Methods

A crossover approach is performed via the contralateral common femoral artery (CFA) using a 4Fr sheath*.* Heparin (2000 IU) is administered to maintain an activated clotting time (ACT) of 150–250 seconds, reducing the risk of thrombus formation during the procedure. As this procedure is a catheter-based therapeutic approach, heparin use is essential to prevent thrombus formation within the catheter, which could otherwise lead to secondary complications. Therefore, ACT is monitored and adjusted as necessary. Excessive prolongation of ACT increases the risk of hemorrhagic complications. To balance thrombotic prevention with bleeding risk, the heparin dosage is minimized to an optimal level. Based on these considerations, an initial heparin dose of 2000 IU is deemed appropriate.

**Fig. 1:**
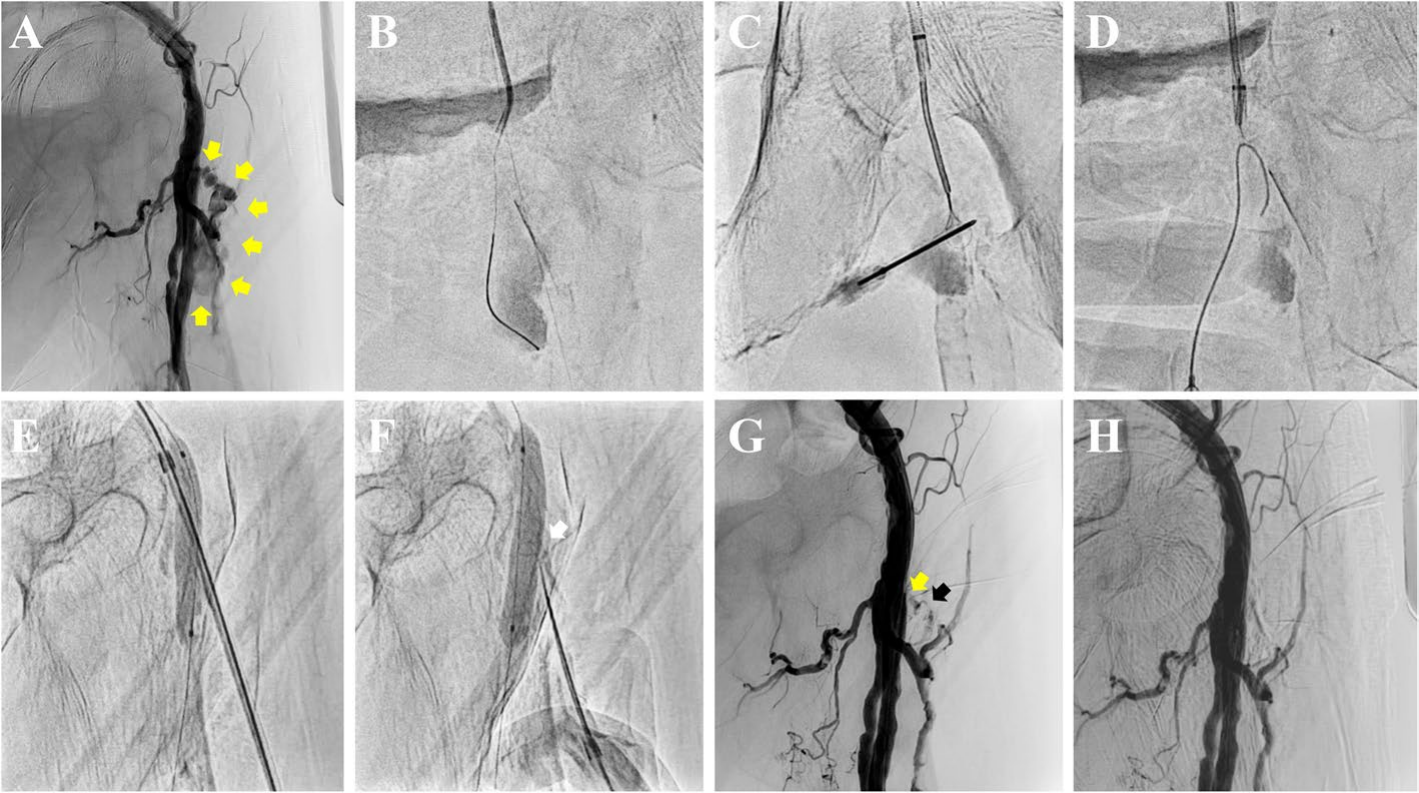
The PseuSeal technique using a snare (PseuSeal Snare). **A** Initial angiography via a contralateral crossover approach revealing a pseudoaneurysm (yellow arrows) in the left common femoral artery (CFA). **B** Advancement of an antegrade system into the pseudoaneurysm cavity. **C** Deployment of a snare device through a 4Fr catheter, followed by retrograde puncture of the snare loop using an 18-gauge needle. **D** Passage of a 0.035-inch guidewire through the needle lumen and subsequent capture with the snare to externalize the guidewire. **E** Retrograde insertion of the ExoSeal-specific sheath over the guidewire from the pseudoaneurysm cavity through its neck into the CFA, followed by balloon inflation to stabilize the system. **F** Deployment of the ExoSeal indicator wire and subsequent withdrawal to the pseudoaneurysm neck (white arrow) to inject a polyglycolic acid (PGA) plug. **G** Angiography showing implanted PGA plug (black arrow) and residual inflow (yellow arrow) into the pseudoaneurysm 5 minutes after plug deployment. **H** Final angiography confirming complete pseudoaneurysm sealing after an additional 5 minutes of balloon hemostasis

**Fig. 2:**
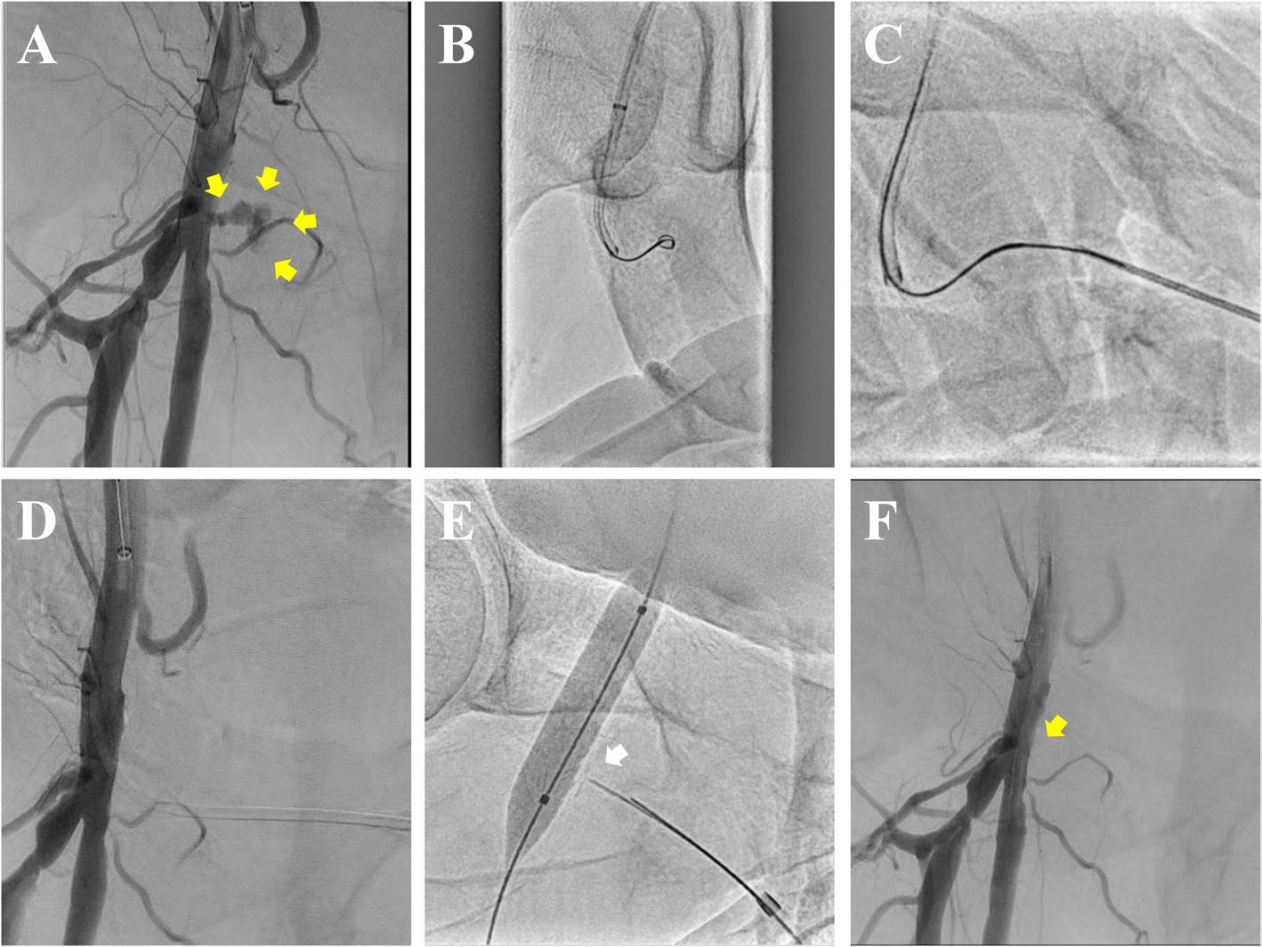
The PseuSeal technique using needle rendezvous (PseuSeal Rendezvous). **A** Initial angiography via a contralateral crossover approach demonstrating a pseudoaneurysm (yellow arrows) at the bifurcation of right common femoral artery (CFA). **B** Advancement of a 0.014-inch guidewire and microcatheter into the pseudoaneurysm cavity, as a 4Fr catheter cannot be advanced. **C** Retrograde puncture of the guidewire using an 18-gauge needle, followed by advancement of the guidewire into the needle lumen to achieve guidewire externalization (needle rendezvous). **D** Advancement of the microcatheter outside the body, then mounting the ExoSeal-specific sheath onto the microcatheter and inserting it into the CFA lumen. **E** Deployment of the ExoSeal indicator wire and subsequent withdrawal to the pseudoaneurysm neck (white arrow) for the delivery of a polyglycolic acid (PGA) plug. **F** Final angiography confirming successful sealing of the pseudoaneurysm (yellow arrow) 5 minutes after plug deployment with balloon hemostasis

A polymer-coated guidewire (Regalia XS 1.0, Asahi Intecc, Japan) and a microcatheter (Corsair PV, Asahi Intecc, Japan) are advanced through the CFA lumen, passing through the pseudoaneurysmal neck into the aneurysmal cavity (Fig. 1A, B). If the 4Fr catheter (Tempo^®^, Cordis, USA) successfully reaches the pseudoaneurysm cavity, a 3.2Fr snare with a loop size of 2–4 mm (EN Snare^®^, Merit Medical Inc., USA) is deployed within the cavity through the catheter. Subsequently, an 18-gauge needle (Terumo, Japan) is inserted retrogradely from outside the body toward the snare loop (Fig 1C). A 0.035-inch guidewire (Radifocus^TM^, Terumo, Japan) is advanced through the needle lumen and captured using the snare. The guidewire is then externalized through the crossover sheath (PseuSeal Snare) (Fig. 1D). The ExoSeal-specific sheath is inserted over the guidewire from the pseudoaneurysm cavity through its neck into the CFA. If the 4Fr catheter cannot be advanced into the aneurysmal cavity, an alternative technique (PseuSeal Rendezvous) is employed. An 18-gauge needle is advanced externally toward the guidewire positioned within the pseudoaneurysm. The guidewire is manipulated into the needle lumen and externalized (needle rendezvous) (Fig 2A–C) [3, 4]. The microcatheter is then advanced externally, and the ExoSeal-specific sheath is mounted onto the microcatheter and inserted into the CFA.

After sheath insertion using either method, a second guidewire is advanced through the crossover sheath into the superficial femoral artery. A semi-compliant balloon is positioned across the pseudoaneurysm ostium within the CFA lumen (Fig. 1E, 2D). The ExoSeal device is inserted retrogradely through the sheath. As the sheath is withdrawn, the balloon is inflated to occlude arterial blood flow. The expanded balloon presses the indicator wire against the vessel wall inside the lumen, ensuring precise deployment of the PGA plug at the pseudoaneurysm neck directly above the aneurysmal ostium (Fig. 1F, 2E). The balloon is deflated after 5 minutes to confirm the absence of blood flow into the pseudoaneurysm (Fig. 2F). If residual inflow is observed (Fig. 1G), the balloon is reinflated for an additional 5 minutes to achieve sealing. The absence of blood flow into the pseudoaneurysm is confirmed, marking the successful completion of the endovascular repair (Fig. 1H).

This approach requires access to the contralateral femoral artery, increasing the risk of pseudoaneurysm at the secondary puncture site. A pre-procedural CFA assessment, minimizing heparin dosage, and administering protamine post-procedure to neutralize heparin are recommended to reduce this complication.

ExoSeal devices are available in sizes compatible with 5-, 6-, and 7-Fr sheaths, with plug diameters of 1.6-, 1.8-, and 2.0 mm, respectively. Given the ExoSeal plug length of 7.0 mm, the maximum theoretically treatable pseudoaneurysm neck width using this technique is approximately 7.0 mm, achieved by positioning the sheath horizontally and deploying the plug directly into the neck. If the neck width exceeds 7.0 mm, the risk of plug displacement and incomplete closure increases, potentially compromising the procedural outcome.

The PseuSeal technique is recommended as an alternative treatment for cases where achieving hemostasis with UGC is challenging, where the risk of complications from thrombin injections in an off-label manner is high, or in anatomically complex cases with significant morbidity and invasiveness associated with surgical repair.

## Results

Five cases of CFA pseudoaneurysm following unsuccessful UGC were treated using the PseuSeal technique. The procedural characteristics are summarized in Table 1. All cases were managed with a 6-Fr ExoSeal. The procedure was successfully completed in all patients without any complications or recurrence during a follow-up period of up to one year. Two representative cases are presented below.

**Table 1:** Case Summary

Case	Age/ Gender	Prior-sheath size (Fr)	Antiplatelet drugs, Anticoagulants	Neck	Aneurysm	Time to PseuSeal (days)	UGC time (min)	PseuSeal method	PseuSeal procedure Time (min)
				width × length (mm)	diameter × height (mm)				
1	87/F	6	Aspirin, Apixaban	3.9 × 7.2	34.4 × 14.7	1	40	Snare	32
2	60/M	7	Aspirin, Clopidogrel	2.8 × 8.0	60.4 × 49.4	3	60	Rendezvous	50
3	61/M	6	Aspirin, Prasugrel	3.2 × 9.1	15.3 × 8.3	2	45	Snare	28
4	62/M	5	Edoxaban	1.5 × 10.3	6.5 × 5.2	7	50	Rendezvous	25
5	73/F	6	Aspirin, Clopidogrel, Warfarin	2.6 × 8.6	23.2 × 11.8	1	120	Rendezvous	32

*Abbreviations: F* female, M male, *UGC* ultrasound-guided compression

### Case 1

An 87-year-old female with chronic limb-threatening ischemia, receiving dual antithrombotic therapy (aspirin and apixaban), developed a pseudoaneurysm at the 6Fr sheath insertion site in the left CFA following endovascular treatment. The pseudoaneurysm was detected on postoperative day 1. UGC for 40 minutes was unsuccessful in achieving closure. Extensive calcification at the pseudoaneurysm site rendered UGTI and surgical repair highly challenging. Therefore, the PseuSeal technique was performed. A 4Fr catheter was successfully advanced into the pseudoaneurysm cavity, completing the PseuSeal Snare. Hemostasis was achieved within 32 minutes without complications. Follow-up Doppler ultrasound at 1 month confirmed complete occlusion of the pseudoaneurysm.

### Case 2

A 60-year-old male on hemodialysis, undergoing dual antiplatelet therapy (aspirin and clopidogrel), developed a pseudoaneurysm in the right CFA after percutaneous coronary intervention with a 7Fr sheath. The pseudoaneurysm was detected 3 days post-procedure. Despite 60 minutes of UGC, closure was not achieved. The pseudoaneurysm was located at the bifurcation of the CFA, making blood flow occlusion via balloon inflation technically challenging. Additionally, balloon-assisted thrombin injection [5] was deemed impractical due to the risk of distal embolization. Given the anatomical complexity, surgical intervention was also considered highly invasive. Therefore, the PseuSeal technique was selected. As a 4Fr catheter could not be advanced into the aneurysm cavity, the PseuSeal Rendezvous was performed. Hemostasis was successfully achieved within 50 minutes without complications. A 1-year Doppler ultrasound confirmed no recurrence.

Figures and supplementary materials illustrate these techniques (Figures 1 and 2; Supplementary Videos 1 and 2).

## Discussion

The PseuSeal technique achieves hemostasis through two primary mechanisms: (1) mechanical occlusion of blood flow by physically sealing the neck of the pseudoaneurysm with the ExoSeal plug, and (2) promoting thrombosis around the plug and within the pseudoaneurysm due to reduced blood flow.

ExoSeal has been reported to have a 92% success rate for femoral sheath closure, with no infections related to ExoSeal [6]. Although rare occurrences of plug migration into the vascular lumen have been noted, our technique minimizes this risk through the adjunctive use of balloon inflation during plug deployment. The PerClose device has also been utilized to treat pseudoaneurysms [7]; however, its use entails the deployment of non-absorbable polypropylene sutures. This material may increase the risk of vascular injury and infection, particularly in cases with fragile tissue at the pseudoaneurysm neck. Therefore, the PseuSeal technique may have less risk of infection and rupture than the PerClose method.

The PseuSeal technique has several limitations: 1) This technique involves the off-label use of ExoSeal, which limits the number of cases and poses challenges in its evaluation and generalizability. 2) All five cases were treated using the 6-Fr ExoSeal device. A 5-Fr sheath may lead to insufficient sealing due to a potential gap between the sheath and the pseudoaneurysm neck, increasing the risk of blood leakage during the procedure. Conversely, a 7-Fr sheath may present a higher risk of injury due to its lager diameter and increased mechanical stress on the pseudoaneurysm and surrounding vessels. Thus, the 6-Fr sheath appears to be the optimal choice for this procedure. However, the feasibility of using alternative sheath sizes remains uncertain and requires further investigation.

## Conclusions

The PseuSeal technique, which involves the off-label use of ExoSeal, offers an alternative for managing pseudoaneurysms in cases where standard approaches are ineffective. However, further studies are necessary to evaluate its long-term efficacy and safety.

## Supplementary Information

Supplementary Material 1: Supplementary Video 1: PseuSeal Snare.

Supplementary Material 2: Supplementary Video 2: PseuSeal Rendezvous.

## Data Availability

The data are available from the corresponding author upon reasonable request.

## Abbreviations

ACT: Activated clotting time
CFA: Common femoral artery
PGA: Polyglycolic acid
UGC: Ultrasound-guided compression
UGTI: Ultrasound-guided thrombin injection
